# Prevalence and Risk Factors of Rubella and Cytomegalovirus Infections Among Pregnant Women in Makkah: Implications for Screening and Vaccination Programs

**DOI:** 10.7759/cureus.57269

**Published:** 2024-03-30

**Authors:** Khalil Mohammed

**Affiliations:** 1 Epidemiology and Medical Statistics, Faculty of Public Health and Health Informatics, Umm Al-Qura University, Makkah, SAU

**Keywords:** holy makkah, risk factors, prevalence, cytomegalovirus, rubella

## Abstract

Introduction

Contracting rubella virus or cytomegalovirus (CMV) while pregnant can lead to severe health issues for both the mother and the unborn child. This study aims to determine the prevalence of these infections in pregnant women and identify associated risk factors.

Methods

A total of 146 pregnant women consented to participate in this research. Data were collected through a detailed questionnaire and blood samples were obtained from each participant. Blood was drawn into vacutainer tubes, and plasma was separated and stored at -20°C for analysis. We utilized specific enzyme-linked immunosorbent assays (ELISA) for immunoglobulin G (IgG) and Immunoglobulin M (IgM) to detect antibodies against rubella and CMV in the plasma samples.

Results

The study revealed that the prevalence rates of IgG antibodies for rubella and CMV were 68.5% and 97.3%, respectively. No samples tested positive for IgM antibodies for either disease. A significant relationship was found between chronic rubella infection and women aged 26 to 35 years (p < 0.05). A significant association was also observed between chronic CMV infection and women with lower income (p < 0.05).

Conclusion

This study confirms the widespread presence of chronic rubella and CMV infections among pregnant women in Makkah. The findings highlight the impact of socioeconomic factors on infection rates and underscore the importance of implementing vaccination programs to mitigate the severity of these infections in pregnant women and protect fetal health.

## Introduction

Rubella virus and cytomegalovirus (CMV) are significant global health concerns, particularly during pregnancy, as both viruses can cause worldwide infections [1]. Primary infections with rubella and CMV can lead to severe complications for pregnant women and their fetuses [2]. The rubella virus, an RNA virus from the Rubivirus genus within the family Matonaviridae, exclusively infects humans, with no evidence of animal reservoirs [3-5]. In contrast, CMV is a member of the Herpesviridae family and the Betaherpesvirinae subfamily [6]. These infections are particularly alarming in pregnant women because they can cause intrauterine infections, potentially leading to fetal death [7,8].

Rubella virus infection during early pregnancy can lead to congenital rubella syndrome, which is characterized by a group of congenital disorders that may cause spontaneous abortion in 65% to 85% of cases [9,10]. Symptoms can be temporary, such as low birth weight, or permanent, including hearing loss and developmental issues like myopia. Other common manifestations are cataracts, glaucoma, deafness, intellectual disability, and congenital cardiac disease [10,11]. CMV infection in pregnant women can lead to a variety of fetal complications, including hepatosplenomegaly, microcephaly, cerebral calcifications, intellectual disabilities, jaundice, chorioretinitis, petechial rash, or multi-organ involvement, especially when the infection occurs in the first trimester [11].

Studies have shown a high prevalence of rubella virus infection among pregnant women in African countries, with seroprevalence rates of Immunoglobulin G (IgG) reaching 97.9% in Nigeria and 65.3% in Sudan. The highest recorded prevalence of Immunoglobulin M (IgM) in the acute phase antibodies was 38.8% in Nigeria, with the lowest at 0.3% in Tanzania. In Asia, Thailand reported a high prevalence of rubella virus of 89.4%, while Laos had a low of 43.6%. In Saudi Arabia, the seroprevalence rate for past infections reached 88.9% in Abha, with no recent infections reported [12-18]. In the United States, the seroprevalence of CMV has ranged from 10% to 30% among populations of lower socioeconomic status and non-white ethnicities [19,20]. In Africa, the seroprevalence of CMV among pregnant women has ranged from 60.6% in Tanzania [21] to 100% in Gambia [22]. High rates were also reported in some Arabic countries, including Yemen [23], and Saudi Arabia [24].

A study conducted in Makkah in 2002, including TORCH (Toxoplasmosis, Other agents, Rubella, CMV, and Herpes simplex) infections, showed that the prevalence rates of rubella and CMV were very high, reaching 93% [25]. Given this context, the research aims to determine the current seroprevalence of rubella and CMV among pregnant women in Makkah City and to identify associated risk factors.

## Materials and methods

This cross-sectional study investigated the prevalence rates of rubella virus and CMV infections among pregnant women in Makkah City, focusing on the acute and chronic stages of these diseases. A total of 146 pregnant women consented to participate in the study. Eligible participants were those who could independently sign the consent form. Participants at different stages of pregnancy and from different age groups regularly attended an antenatal care clinic for pregnancy follow-ups. Most of the participants were based in Holy Makkah, a densely populated area in western Saudi Arabia, known for Al-Masjid Al-Haram, which attracts more than 25 million visitors annually, including pilgrims and tourists.

The Biomedical Research Ethics Committee at Umm Al-Qura University approved the study (No. HAPO-02-K-012-2021-02-545). We obtained written consent from each participant and collected demographic, socioeconomic, and health-related data through a questionnaire. This included information on age, education, occupation, residence, income, number of previous pregnancies, previous abortions, and stillbirths.

We collected a 5 ml venous blood sample from each participant into a tube containing ethylenediaminetetraacetic acid. After centrifugation at 10,000 g, we separated the plasma and stored it at -20°C for further analysis. We tested the plasma samples for anti-rubella virus IgG and IgM and for anti-CMV IgG and IgM using commercial kits (Human Co, Germany; https://www.human.de/), following the manufacturer's guidelines. An enzyme-linked immunosorbent assay (ELISA) reader quantified the results, with positive and negative controls included on the same plate.

Statistical analysis

We used IBM SPSS Statistics for Windows, Version 25 (Released 2017; IBM Corp., Armonk, New York, United States) for data analysis. The Chi-square test assessed the association between serology results and participant characteristics.

## Results

This study included 146 pregnant women, predominantly residing in Makkah City, accounting for 116 (79.5%) of participants. Their ages spanned from 18 to 45 years, with the most represented age group being between 25 and 35 years old, which included 78 participants (53.4%). The educational level was high among the participants, with 78 being graduates (53.4%), and employment was noted in 22 cases (15.1%). Approximately 58 of the women (39.7%) had been married for less than five years. The distribution of pregnancy stages among the participants showed that most of them were in their first trimester (n=68; 46.6%); 38 (26%) had no children; a history of abortion was reported in 66 (45.2%), and pre-term labor occurred in 12 cases (8.2%).

Our study aimed to assess the seroprevalence of rubella and CMV in pregnant women within Makkah City and to identify associated risk factors. We did not detect any acute infections. However, chronic infections, as evidenced by the presence of IgG antibodies, were prevalent in 142 cases (97.3%) for CMV and 100 cases (68.5%) for rubella. It is noteworthy that there was statistically a significant relationship between the prevalence of chronic rubella infection in women aged 25 to 35 years and CMV infection and lower income levels, as detailed in Table 1.

**Table 1:** Prevalence of acute and chronic rubella and cytomegalovirus +ve: Positive; -ve: Negative

Antibodies	Rubella virus		Total	Cytomegalovirus		Total
	+ve	-ve		+ve	-ve	
Acute IgM	0 (0%)	146 (100%)	146	0 (0%)	146 (100%)	146
Chronic IgG	100 (68.5%)	46 (31.5%)	146	142 (97.3%)	4 (2.7%)	146

The questionnaire-based data collection highlighted significant associations between chronic infections and various demographic, socioeconomic, and health-related factors. Chronic rubella infection was significantly more prevalent in the 25-35-year age group (p < 0.05), as shown in Table 2. This age-specific trend was not observed for chronic CMV infections. Pregnant women living in Makkah exhibited higher seroprevalence rates for rubella (61.6%) and CMV (83.6%) compared to those residing outside Makkah. Education level appeared to be a factor, as graduates showed a higher susceptibility to both infections 37.0% and 52.1% for rubella and CMV, respectively. Compared to their employed counterparts, housewives were more susceptible to both diseases.

**Table 2:** Risk factors associated with chronic infection of rubella +ve: Positive; -ve: Negative; X2: The value of Chi squire

Variable	Branch of variable	Target Groups		X^2^	p-value
		IgG +ve	IgG -ve		
Age groups (Years)					
	18-25	18 (12.3%)	2 (1.4%)	7.957	0.047
	26-35	52 (35.6%)	36 (24.7%)		
	36-45	30 (20.5%)	6 (4.1%)		
	>45	0 (0.0%)	2 (1.4%)		
Resident					
	Inside Makkah	90 (61.6%)	36 (24.7%)	1.836	0.161
	Outside Makkah	10 (6.8%)	10 (6.8%)		
Education					
	Uneducated	2 (1.4%)	0 (0.0%)		
	Primary & Intermediate	14 (9.6%)	16 (11.0%)	5.618	0.132
	Secondary	30 (20.5%)	6 (4.1%)		
	Graduate	54 (37.0%)	24 (16.4%)		
Occupational Status					
	Working	20 (13.7%)	2 (1.4%)	3.016	0.077
	Not working	80 (54.8%)	44 (30.1%)		
Income					
	3000-5000	42 (29.2%)	26 (18.1%)		
	5000-10000	38 (26.4%)	16 (11.1%)	1.644	0.44
	10000-15000	9 (12.5%)	4 (2.8%)		
Duration of married					
	1-5	42 (28.8%)	16 (11.0%)		
	5-10	34 (23.3%)	20 (13.7%)	0.69	0.876
	10-15	16 (11.0%)	6(4.1%)		
	>15	8 (5.5%)	4 (2.7%)		
Number of previous pregnancies					
	None	16 (11.0%)	10 (6.8%)		
	1-3	40 (27.4%)	16 (11.0%)	0.597	0.897
	3-6	34 (23.3%)	14 (9.6%)		
	>6	10 (6.8%)	6 (4.1%)		
Number of previous deliveries					
	None	28 (19.2%)	10 (6.8%)		
	1-3	46 (31.5%)	28 (19.2%)	2.162	0.54
	3-6	24 (16.4%)	6 (4.1%)		
	>6	2 (1.4%)	2 (1.4%)		
Second wife					
	Yes	6 (4.1%)	6 (4.1%)		
	No	94 (64.4%)	40 (27.4%)	1.036	0.278
Abortion					
	Yes	42 (29.2%)	24 (16.7%)		
	No	56 (38.9%)	22 (15.3%)	0.547	0.313
Pre-term labor					
	Yes	4 (2.7%)	8 (5.5%)		
	No	96 (65.8%)	38 (26.0%)	3.745	0.074

The analysis also revealed a significant association between CMV infection and lower income (p < 0.05), whereas rubella more frequently affected women with middle incomes. The association between the length of marriage and infection rates was also noteworthy; rubella was more prevalent among women in the early years of marriage, while CMV showed higher rates in both early and middle years of marriage. Women with one to three previous pregnancies demonstrated similar prevalence rates for both infections, as detailed in Tables 2, 3.

**Table 3:** Risk factors associated with chronic infection of cytomegalovirus +ve: Positive; -ve: Negative;  X2: The value of Chi squire

Variable	Branch of variable	Target Groups		X^2^	p-value
		IgG +ve	IgG -ve		
Age groups					
	18-25	18 (12.3%)	2 (1.4%)	2.549	0.466
	26-35	86 (58.9%)	2 (1.4%)		
	36-45	36 (24.7%)	0 (0.0%)		
	>45	2 (1.4%)	0 (0.0%)		
Resident					
	Inside Makkah	122 (83.6%)	4 (2.7%)	0.836	0.568
	Outside Makkah	20 (14.1%)	0 (0.0%)		
Education					
	Uneducated	2 (1.4%)	0 (0.0%)		
	Primary & Intermediate	30 (20.5%)	0 (0.0%)	0.991	0.803
	Secondary	34 (23.3%)	2 (1.4%)		
	Graduate	76 (52.1%)	2 (1.4%)		
Occupational Status					
	Working	20 (13.7%)	2 (1.4%)	1.961	0.280
	Not working	122 (83.6%)	2 (1.4%)		
Income					
	3000-5000	68 (47.2%)	0 (0.0%)		
	5000-10000	52 (37.5%)	0 (0.0%)	11.408	0.003
	10000-15000	18 (12.5%)	4 (2.8%)		
Duration of married					
	1-5	54 (37.0%)	4 (2.7%)		
	5-10	54 (37.0%)	0 (0.0%)	3.120	0.373
	10-15	22 (15.5%)	0 (0.0%)		
	>15	12 (5.5%)	0 (0.0%)		
Number of previous pregnancies					
	None	24 (16.4%)	2 (1.4%)		
	1-3	54 (37.0%)	2 (1.4%)	2.171	0.538
	3-6	48 (32.9%)	0 (0.0%)		
	>6	16 (11.0%)	0 (0.0%)		
Number of previous deliveries					
	None	34 (23.3%)	4 (2.7%)		
	1-3	74 (50.7%)	0 (0.0%)	5.844	0.119
	3-6	30 (20.5%)	0 (0.0%)		
	>6	4 (2.7%)	0 (0.0%)		
Second wife					
	Yes	12 (8.2%)	0 (0.0%)		
	No	130 (89.0%)	4 (2.7%)	0.184	0.841
Abortion					
	Yes	64 (44.4%)	2 (1.4%)		
	No	76 (52.8%)	2 (1.4%)	0.014	0.905
Pre-term labor					
	Yes	12 (8.2%)	0 (0.0%)	0.184	0.668
	No	130 (89.0%)	4 (2.7%)		

## Discussion

Our study corroborates previous research conducted among similar populations in the same region. A previous study reported the prevalence of chronic rubella to be 88.9% [18], compared to 68.5% in our current findings. Notably, our research did not detect any acute rubella infections among participants in alignment with the previous study [18]. Furthermore, research from China highlighted severe pregnancy outcomes in patients infected with TORCH, including congenital malformations (12.9%), abortions (31.8%), premature labor (8.2%), and infant deaths (9.4%) [26]. Similarly, our study identified no acute infections by CMV or rubella, with chronic infections identified through IgG antibodies being more prevalent in CMV (97.3%) than in rubella (68.5%). This is consistent with a study from the Jizan province of Saudi Arabia, which reported a CMV IgG positivity rate of 93.1% among pregnant women [27].

Our findings also demonstrated a significant association between chronic rubella infections and the age group of 26 to 35 years, indicating higher susceptibility (p < 0.05). This is supported by a study in Al Khobar, Saudi Arabia, showing a high seroprevalence rate of herpes simplex virus-1 IgG antibodies (93.2%) [27]. Studies in Makkah and Riyadh on Toxoplasma gondii reported IgM positivity rates and associated adverse obstetric outcomes with past infections, suggesting a link between TORCH infections and poor pregnancy outcomes [28].

A pivotal finding of our research is the association between chronic CMV infection and lower income, aligning with previous studies that identified an inverse relationship between socioeconomic status and chronic diseases [29]. This suggests that lower socioeconomic status significantly increases the risk of infections, warranting further investigation into the biophysical processes linking socioeconomic status to health outcomes.

Our results underscore the prevalence of rubella and CMV infections in the Kingdom of Saudi Arabia, highlighting the potential for these infections to cause congenital infections and other complications. The findings advocate for intermittent screening among pregnant women to prevent TORCH complications, which could lead to neonatal morbidity and pregnancy-related complications [18].

Our study had several important limitations related to time and resource constraints, which may affect the depth and scope of our analysis. Time constraints may require adopting less comprehensive data collection methods or reducing the study duration, potentially limiting the richness of the collected data. Additionally, the sample size and representativeness could restrict our ability to generalize findings to all pregnant women in Makkah or other regions, as the sampling method may not have captured a sufficiently broad range of the population. The cross-sectional design of our study limits our ability to infer a causal relationship between the observed factors and incidence rates. Furthermore, reliance on self-reported data might introduce bias due to inaccurate recall or social desirability effects, which may skew information related to health history and exposure to risk factors.

Our focus on a specific geographic area also means that the results may not apply to populations in different settings, where demographic and health system differences may influence infection rates. The diagnostic methods employed, primarily the detection of IgG antibodies, may not be able to capture the full spectrum or stages of infection, given the tests' sensitivity and specificity limitations. The absence of a control group hinders our ability to fully assess the impact of pregnancy on infection rates compared to non-pregnant women or those from varied backgrounds. The study also may not have taken into account all potential confounding factors that could influence the prevalence of infections, such as vaccination history, previous exposure, and other environmental or genetic variables. Lastly, limitations in data collection techniques and the analytical methods used might have affected the findings, suggesting that a more nuanced statistical analysis could reveal additional insights. Acknowledging these limitations is crucial for interpreting the study's results and underscores the need for further research to address these gaps.

## Conclusions

The research aims to determine the current seroprevalence of rubella and CMV among pregnant women in Makkah City. This study confirms the widespread chronic infections of rubella and CMV among pregnant women in Makkah; the IgG antibodies against rubella are protective for the fetus. Therefore, pregnant women with IgG antibodies were out of the risk zone. The findings highlight the impact of socioeconomic factors, particularly income level, on the prevalence of these infections. To mitigate the severity of these diseases in pregnant women and their fetuses, it is recommended to implement vaccination programs. 

## Appendices

**Table 4:** Questionnaire: Prevalence of rubella and cytomegalovirus among pregnant women in Makkah Al Mukarramah

18-25	
26-35	
36-45	
>45	

Inside Makkah	
Outside Makkah	

Uneducated	
Primary & Intermediate	
Secondary	
Graduate	

Working	
Not working	

3000-5000	
5000-10000	
10000-15000	

1-5	
5-10	
10-15	
>15	

None	
1-3	
3-6	
>6	

None	
1-3	
3-6	
>6	

Yes	
No	

Yes	
No	

Yes	
No	
